# Nurses and the administration of Adrenaline in anaphylactic shock following the application of IV medicine

**DOI:** 10.1186/1757-7241-23-S1-A45

**Published:** 2015-07-16

**Authors:** Rudolf Scheller, Stefan Posth

**Affiliations:** 1 Department of Geriatric Medicine, OUH Odense University Hospital, Odense, Denmark; 2 Department of Emergency Medicine, OUH Odense University Hospital, Odense, Denmark

## 

"We recommend first-line treatment with intramuscular adrenaline before instituting other interventions as adrenaline is still underutilized in anaphylaxis although it is potentially lifesaving."

EAACI Guidelines Anaphylaxis 2014

## How is reality in a Danish university hospital?

At Odense University Hospital, nurses take part in a simulation training in critical patient care for younger doctors. One of the scenarios used is anaphylactic shock after the application of IV antibiotics. 49 out of 50 nurses realized within the first two minutes, that the simulation patient suffered from anaphylaxis. 48 out of 50 nurses stopped the IV with the antibiotic. Often, oxygen and IV NaCl was administered, and thereafter the doctor, who was five minutes away was called. Only two out of 50 nurses primarily injected adrenalin according to the hospital guideline.

Because of this, in 48 out of 50 cases adrenalin was given after more than five minutes. According to Pumphrey (2000), the median for a deadly anaphylactic reaction after the IV administration of medication is five minutes. Adrenalin is according to EAACI Guidelines 2014 and ERC Guidelines 2010 the primary treatment for anaphylactic shock and the only treatment that is lifesaving in such a situation.

## Conclusion

It is necessary for all nurses to receive training in anaphylactic shock, including the primary injection of adrenalin in order to secure immediate treatment in this critical condition.

This training could be linked to resuscitation training.

